# BayesRVAT enhances rare-variant association testing through Bayesian aggregation of functional annotations

**DOI:** 10.1101/gr.280689.125

**Published:** 2025-12

**Authors:** Antonio Nappi, Liubov Shilova, Theofanis Karaletsos, Na Cai, Francesco Paolo Casale

**Affiliations:** 1 Institute of AI for Health, Helmholtz Zentrum München – German Research Center for Environmental Health, 85764 Neuherberg, Germany;; 2 Helmholtz Pioneer Campus, Helmholtz Zentrum München – German Research Center for Environmental Health, 85764 Neuherberg, Germany;; 3 School of Computation, Information and Technology, Technical University of Munich, 85748 Garching, Germany;; 4 AI Resident, Chan Zuckerberg Initiative, Redwood City, California 94063, USA;; 5 Friedrich-Alexander-Universität Erlangen-Nürnberg, 91054 Erlangen, Germany;; 6 Chan Zuckerberg Initiative, Redwood City, California 94063, USA;; 7 TUM School of Medicine and Health, Technical University of Munich and Klinikum Rechts der Isar, 81675 Munich, Germany;; 8 Department of Biosystems Science and Engineering, ETH Zürich, 4056 Basel, Switzerland

## Abstract

Gene-level rare variant association tests (RVATs) are essential for uncovering disease mechanisms and identifying therapeutic targets. Advances in sequence-based machine learning have generated diverse variant pathogenicity scores, creating opportunities to improve RVATs. However, existing methods often rely on rigid models or single annotations, limiting their ability to leverage these advances. Here, we introduce BayesRVAT, a Bayesian rare variant association test that jointly models multiple annotations. By specifying priors on annotation effects and estimating gene- and trait-specific posterior burden scores, BayesRVAT flexibly captures diverse rare-variant architectures. In simulations, BayesRVAT improves power while maintaining calibration. In UK Biobank analyses, it detects 10.2% more blood-trait associations and reveals novel gene–disease links, including *PRPH2* with retinal disease. Integrating BayesRVAT within omnibus frameworks further increases discoveries, demonstrating that flexible annotation modeling captures complementary signals beyond existing burden and variance-component tests.

Understanding the role of rare genetic variants is crucial for uncovering disease mechanisms and identifying potential therapeutic targets. Prioritizing variants with lower frequencies and potential functional impact, rare variant analyses tend to provide a more interpretable approach compared to common variant studies (Cirulli et al. 2020; McCaw et al. 2023).

Gene-based rare variant association tests (RVATs) aim to identify genes influencing traits of interest. Traditionally, gene-based RVATs have been performed using burden tests, which aggregate likely deleterious variants into a gene burden score and then regress these scores against trait values across individuals in a formal gene-level association test (Li and Leal 2008; Madsen and Browning 2009; Brandes et al. 2020; Backman et al. 2021; Jurgens et al. 2022; Karczewski et al. 2022). Likely deleterious variants within a gene are typically identified based on consequence annotations (e.g., protein-truncating variants tend to have stronger effects than missense variants) (McLaren et al. 2016) and functional effect prediction scores such as PolyPhen-2 and SIFT (Kumar et al. 2009; Adzhubei et al. 2010; Karczewski et al. 2022). More recently, machine learning models trained on biological sequences to predict functional and structural properties have expanded the availability of variant pathogenicity prediction scores (Zhou and Troyanskaya 2015; Sundaram et al. 2018; Jaganathan et al. 2019; Ghanbari and Ohler 2020; Brandes et al. 2023; Cheng et al. 2023; Wagner et al. 2023), providing new opportunities for improved variant prioritization.

Recent burden test models can incorporate multiple variant annotations directly implementing the concept of an *allelic series*, where increased likelihoods of gene disruptions correspond to stronger phenotypic effects (McClintock 1944; Musunuru and Kathiresan 2019). For instance, COAST weights variants based on their predicted deleteriousness (McCaw et al. 2023), whereas DeepRVAT employs a data-driven approach to learn aggregation functions from multiple annotations using a neural network (Clarke et al. 2024). Despite these improvements, a unified framework that jointly models multiple annotations while allowing flexible, gene- and trait-specific aggregation is still missing.

To address this, we present BayesRVAT, a Bayesian RVAT framework that flexibly aggregates variant effects using multiple annotations. Inspired by the concept of allelic series, BayesRVAT models variant effects as a function of multiple annotations and aggregates them in a gene- and trait-specific manner through Bayesian inference, capturing how different annotations shape gene burden. To compute association *P*-values within this Bayesian framework, we introduce an approximate likelihood ratio test. We validate BayesRVAT through simulations and analyses of quantitative and binary traits from the UK Biobank, demonstrating improved performance over existing gene-level RVAT strategies.

## Results

### Bayesian aggregation for rare variant association testing

**Figure 1.**
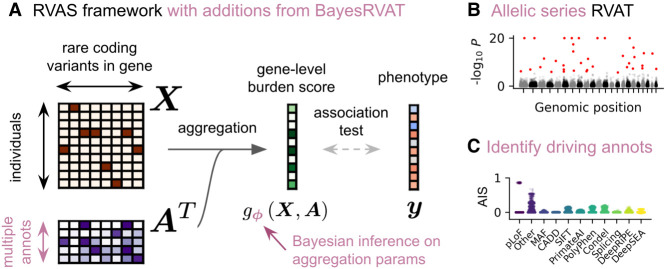
Overview of the BayesRVAT framework. (*A*) In rare variant association tests (RVATs), rare variants *X* and their annotations *A* are aggregated into a gene burden score, which is tested for association with the phenotype *y*. BayesRVAT explicitly introduces aggregation function *g*_*φ*_(*X*, *A*) and a prior over aggregation parameters *ϕ*. (*B*) BayesRVAT enables scalable gene burden testing accounting for multiple annotations. (*C*) It also provides annotation importance scores (AIS) for each analyzed gene–trait pair.

Gene-level burden tests aggregate the effects of rare variants within a gene into a single burden score, which is then tested for association with the trait of interest. Formally, given trait values *y* ∈ *R*^*N*^ for *N* individuals, genotype matrix *X* = [*x*_1_, …, *x*_*N*_]^*T*^ ∈ *R*^*N*×*S*^ for *S* rare variants, annotation matrix *A* ∈ *R*^*S*×*L*^ encoding *L* functional annotations, and covariate matrix *F* ∈ *R*^*N*×*K*^ for *K* factors (e.g., age, sex, and leading genetic principal components), gene-level burden tests consider the following linear model (Fig. 1A):

$$y=F\alpha +g(X,A)\text{β}+\psi ,\;\mathrm{with}\;\psi ∼N(0,\;\sigma_{n}^{2}I_{N}),$$

where *g*(*X*, *A*) = [*g*(*x*_1_, *A*), …, *g*(*x*_*N*_, *A*)] ∈ *R*^*N*×1^ is a function aggregating variant effects for each individual based on annotations *A* in a gene-level burden score, *α* ∈ *R*^*K*^ is the vector of covariate effects, *β* is the effect size of the burden score, and $\sigma_{n}^{2}$ is the residual variance. For example, *g*(*X*, *A*) could be the sum of putative loss-of-function (pLoF) variants within a gene for each individual (Cirulli et al. 2020). Within this framework, statistical association between the gene burden score and the phenotype is assessed by testing whether *β* ≠ 0.

In BayesRVAT, we enhance flexibility by making the aggregation function *g*_*φ*_(*X*, *A*) dependent on parameters *ϕ*, which describe how variants are integrated into a burden score based on their annotations (Fig. 1A). Specifically, we use a linear model with saturation

$$g_{\phi }(X,\;A)=\sigma (XA\phi -b_{0}),$$

where annotations *A* are processed such that higher values correspond to more deleterious effects (Methods), *b*_0_ shifts the input such that individuals without rare variants have scores close to zero, and the sigmoid function σ accounts for saturation effects, where additional variants do not further increase the burden once the gene function is already lost. We introduce priors on *ϕ* to reflect biological expectations while modeling uncertainty: We introduce strong effect priors for pLoF variants, while considering weaker effect and higher variance priors for other annotations (Methods; Supplemental Fig. S1).

We employ variational inference to estimate posterior distributions over ϕ and estimate model parameters for each gene–trait pair (Kingma and Welling 2014; Ranganath et al. 2014; Rezende et al. 2014; Methods). After the estimation step, BayesRVAT provides a gene-level association *P*-value (Fig. 1B; Methods) and annotation importance scores (AISs), which quantify the extent to which specific annotations drive the association for the analyzed gene–trait pair (Fig. 1C; Methods).

### Simulations

We evaluated BayesRVAT using simulated data from unrelated individuals in the UK Biobank (UKB) cohort (Methods). Briefly, we simulated gene-level genetic effects from real variant data using a saturated additive model and varied key parameters, including sample size, variance explained by the burden score, and the number of contributing annotations (Methods). For variant annotations, we considered 25 features, including three derived from variant consequences, allele frequency, five functional impact scores, two splicing prediction scores, eight RNA-binding propensities, and six regulatory annotations (Methods).

**Figure 2.**
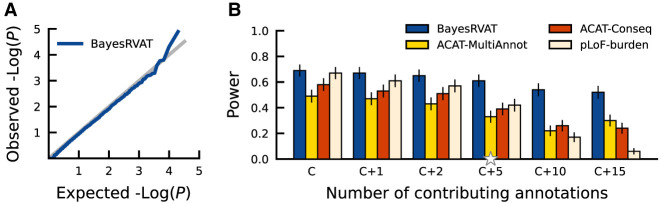
Evaluation of calibration and power in BayesRVAT using synthetic data. (*A*) QQ plot assessing the calibration of *P*-values from BayesRVAT on synthetic data generated under the null model with no genetic effects. (*B*) Statistical power comparison between BayesRVAT, ACAT-MultiAnnot, ACAT-Conseq, and pLoF-burden test, across varying numbers of contributing continuous annotations: simulating only effects from pLoF and missense consequences (C), and considering additional effects from 1 (C+1), 2 (C+2), 5 (C+5), 10 (C+10), and 15 continuous annotations (C+15) (Methods). Power is measured at the exome-wide significance threshold of *P* < 2.5 × 10^−6^, computed over 100 replicates for each scenario. Stars on the *x*-axis indicate default parameter values, which were held constant when varying other parameters.

First, we assessed the statistical calibration of BayesRVAT when simulating under a null model with no genetic effects, which yielded calibrated *P*-values across different simulated sample sizes (Fig. 2A; Supplemental Fig. S2). Next, we compared the statistical power of BayesRVAT against commonly used burden tests, including classical pLoF burden testing; ACAT-Conseq, which aggregates separate tests for pLoF, missense, and other nonsynonymous variants (ACAT-Conseq) (Methods), corresponding to the basic allelic series burden test in McCaw et al. (2023); and ACAT-MultiAnnot, which aggregates separate tests for each of the 25 analyzed annotations (Methods), similar to the method in Li et al. (2020). BayesRVAT consistently outperformed alternative burden tests, sustaining higher power in more complex genetic architectures with increasing contributing annotations while remaining robust to noninformative annotations (Fig. 2B), suggesting that its joint modeling of annotations within an allelic series prior enables effective aggregation of informative contributions across features. BayesRVAT also retained higher power when we simulated causal effects deviating from the allelic-series assumption by introducing annotation-independent, variant-level random effects (Supplemental Fig. S3). The superior performance of BayesRVAT was maintained across varying sample sizes, levels of variance explained by the burden score (Supplemental Fig. S4), and in applications to binary traits, where we confirmed well-calibrated *P*-values and superior power (Methods; Supplemental Figs. S5, S6).

### Analysis of blood biomarkers

**Figure 3.**
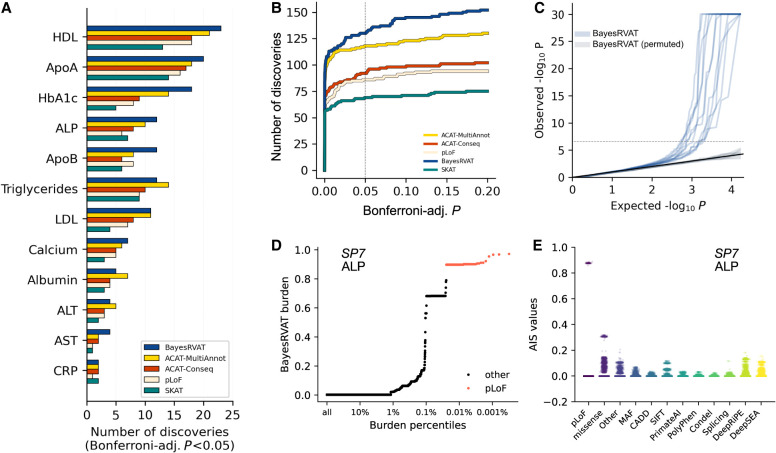
Analysis of blood biomarkers in the UK Biobank. (*A*) Number of significant gene–trait associations (Bonferroni-adjusted *P* < 5 × 10^−2^) discovered by BayesRVAT, ACAT-MultiAnnot, ACAT-Conseq, and pLoF burden tests for each analyzed blood trait. (*B*) Cumulative number of discoveries at varying Bonferroni-adjusted significance thresholds. (*C*) QQ plot showing the distribution of *P*-values from BayesRVAT in real data and under a null with permuted genotype data, confirming well-calibrated *P*-values. (*D*) Burden scores learned by BayesRVAT for *SP7* and ALP across burden percentiles, showing individuals carrying pLoF mutations in red. (*E*) Annotation importance scores (AIS) from BayesRVAT for the association between *SP7* and ALP, which highlights contributions from missense, DeepRiPE, and DeepSEA annotations.

We applied BayesRVAT and alternative burden test strategies to analyze 12 blood traits from the UKB cohort, considering the same set of 25 annotations used in simulations (Methods). BayesRVAT identified a greater number of significant gene–trait associations compared to other methods (130 for BayesRVAT vs. 118 for ACAT-MultiAnnot, 92 for ACAT-Conseq, and 86 for pLoF; Bonferroni-adjusted *P* < 5 × 10^−2^) (Fig. 3A,B; Supplemental Fig. S7), also showing well-calibrated *P*-values under genotype permutation tests (Fig. 3C). BayesRVAT consistently outperformed ACAT-MultiAnnot (Fig. 3B; Supplemental Fig. S7), except when allelic-series assumptions were violated, for example, when pLoF effects are weaker than the effects of other annotations (Supplemental Fig. S8). Notably, compared to the widely used SKAT variance component and SKAT-O optimal tests (Wu et al. 2011; Lee et al. 2012), BayesRVAT also identified more associations (Supplemental Fig. S9).

We further confirmed the benefits of BayesRVAT's pLoF-dominant prior empirically. Compared to a flat prior weighting all consequence-based annotations equally, it yielded ∼15% more associations at Bonferroni-adjusted *P* < 0.05 (Supplemental Fig. S10).

Among the associations uniquely identified by BayesRVAT, several showed strong biological relevance (Fig. 3D,E; Supplemental Fig. S11). For instance, BayesRVAT uniquely detected an association between *SP7* and alkaline phosphatase (ALP). SP7 is a transcription factor, which was shown before to regulate the expression of *ALP* (Yoshida et al. 2012; Lui et al. 2022). In this case, BayesRVAT's burden score assigned higher weight to annotations beyond loss-of-function mutations (Fig. 3D), with AIS scores indicating contributions from missense, DeepRiPE, and DeepSEA annotations (Fig. 3E).

Finally, to assess whether BayesRVAT provides complementary signals to existing omnibus tests, we integrated it into the STAAR omnibus framework (Li et al. 2020). The resulting combined test identified more gene–trait associations than STAAR-O alone (182 vs. 171) (Supplemental Fig. S12), indicating that BayesRVAT contributes additional signals beyond other components.

### Application to disease traits

We applied BayesRVAT to analyze eight disease traits with binary notations: type 2 diabetes, atrial fibrillation, coronary artery disease, asthma, hypertension, age-related macular degeneration (AMD) and other retinal diseases, glaucoma, and cataract (Methods). For comparison, we also evaluated logistic regression-based burden tests using pLoF, ACAT-Conseq, and ACAT-MultiAnnot.

**Figure 4.**
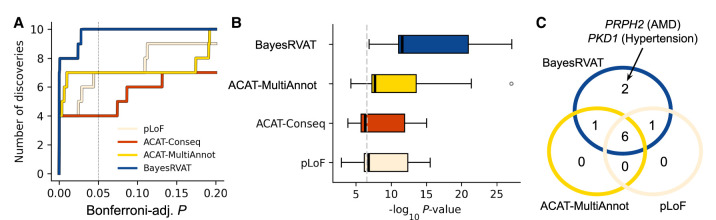
BayesRVAT outperforms alternative burden tests in disease trait analyses. (*A*) Step function showing the cumulative number of significant gene–trait associations as a function of Bonferroni-adjusted *P*. BayesRVAT (blue) consistently identifies more associations than pLoF (beige), ACAT-Conseq (red), and ACAT-MultiAnnot (yellow) across all thresholds. (*B*) Distribution of *P*-values for the nine significant associations identified across all methods (Bonferroni-adjusted *P* < 0.05 for at least one method). (*C*) Venn diagram illustrating the overlap of significant gene–trait associations detected by each method. BayesRVAT recovers all signals found by other methods and uniquely identifies associations between *PKD1* and hypertension, and between *PRPH2* and AMD and other retinal diseases.

BayesRVAT consistently identified more associations than alternative burden tests, detecting 10 significant gene–trait pairs (Bonferroni-adjusted *P* < 0.05) compared to seven for ACAT-MultiAnnot, four for ACAT-Conseq, and seven for pLoF (Bonferroni-adjusted *P* < 5 × 10^−2^) (Fig. 4A; Supplemental Table S1), underscoring its sensitivity. Furthermore, at these loci, BayesRVAT assigned stronger statistical evidence to associations, as reflected in the distribution of *P*-values (Fig. 4B).

BayesRVAT uniquely identified two associations not detected by any other method (Fig. 4C). The first is an association between *PKD1* and hypertension, which can be detected at larger sample sizes (Karczewski et al. 2022). The second is a link between *PRPH2* and AMD and other retinal diseases. *PRPH2* encodes a structural protein in the outer segments of retinal photoreceptors (Kalaw et al. 2025), and multiple *PRPH2* variants have been implicated in retinal conditions (AlAshwal et al. 2025).

## Discussion

In this work, we introduce BayesRVAT, a flexible Bayesian framework for rare variant association testing that integrates multiple functional annotations to improve gene–trait association discovery. By modeling how different annotations contribute to gene disruption and ultimately affect phenotype, BayesRVAT models allelic series, enabling a data-driven aggregation of effects for each analyzed gene–phenotype pair. This is important because different annotations may contribute to gene function disruption in ways that vary across genes and phenotypes (Ritchie and Flicek 2015).

Importantly, the Bayesian allelic series framework in BayesRVAT is particularly relevant given the rapid expansion of machine learning models trained on biological sequences to predict protein structure (Cheng et al. 2023; Gao et al. 2023), gene expression (Avsec et al. 2021; Linder et al. 2025), splicing (Jaganathan et al. 2019; Wagner et al. 2023), or using self-supervised learning approaches (Rives et al. 2021; Nguyen et al. 2024; Benegas et al. 2025; Dalla-Torre et al. 2025). These models have generated large-scale variant pathogenicity estimates, necessitating new methods that integrate these predictions within allelic series models, a gap addressed by BayesRVAT. We note that concurrent work (Das et al. 2025) has also proposed a Bayesian rare variant test integrating annotations, underscoring the timeliness of this research. Conceptually, BayesRVAT can be viewed as an extension of the standard pLoF burden test: Its priors strongly upweight pLoF variants, while allowing other annotations to flexibly contribute to gene disruption in a gene- and trait-specific manner.

BayesRVAT consistently outperformed conventional burden tests, achieving higher power when genetic effects align with its allelic series assumptions. In real data applications, BayesRVAT detected 10.2% more significant associations in blood biomarker analyses and uncovered additional gene–trait associations in disease studies missed by other methods. For example, BayesRVAT uniquely identified the association between *SP7* and alkaline phosphatase, consistent with the *SP7*'s role in *ALP* expression (Yoshida et al. 2012; Lui et al. 2022). It also detected associations between *EPB42* and glycated hemoglobin (Supplemental Fig. S11), in agreement with recent findings linking *EPB42* variants to glycemic traits (Kim et al. 2022), and between *NPC1L1* and apolipoprotein B (Supplemental Fig. S11), reflecting its impact on lipid transport and metabolism (Jia et al. 2011). In the disease trait GWAS, BayesRVAT uniquely linked *PRPH2* to AMD and other retinal diseases. Previously associated with retinitis pigmentosa and pattern dystrophies (AlAshwal et al. 2025), *PRPH2* may contribute to AMD susceptibility through mechanisms affecting photoreceptor stability and function.

The Bayesian burden test implemented in BayesRVAT increases discoveries when integrated within omnibus frameworks, indicating that modeling allelic series through functional annotations captures complementary signals not fully represented by current burden, variance-component, and single-variant tests.

BayesRVAT is not free of limitations. Although it has been optimized for large biobank-scale data sets, with run time increasing linearly with cohort size (Methods; Supplemental Fig. S13), it remains more computationally demanding than simple burden tests due to the use of variational inference. The current formulation assumes additive contributions of annotations, but its modular design facilitates future extensions to model annotation interactions, more flexible modeling of allele-frequency dependencies, or embeddings from self-supervised DNA and protein models (Rives et al. 2021; Nguyen et al. 2024; Benegas et al. 2025; Dalla-Torre et al. 2025). When annotations exhibit collinearity, AIS values may not fully disentangle their individual contributions, and interpretation should therefore focus on the collective importance of correlated annotation groups—for example, by jointly evaluating the importance of each group or by reducing redundancy during preprocessing (e.g., using the first principal components of each group as composite annotations [Li et al. 2020]). Whereas BayesRVAT can be applied to whole-genome sequencing data by leveraging recent advances in regulatory variant effect prediction (Benegas et al. 2025; Hölzlwimmer et al. 2025)—for example, through sliding-window strategies (Morrison et al. 2017) or gene-based masks defined by regulatory elements (Li et al. 2022)—its performance in this context will require further validation. Finally, whereas our analyses focused on unrelated European individuals, BayesRVAT is compatible with upstream approaches accounting for relatedness and population structure (e.g., REGENIE step 1 [Mbatchou et al. 2021]) and can be readily integrated into distributed or cloud-based biobank pipelines.

## Methods

### A Bayesian framework for RVAT

#### The burden test framework

Gene-level burden testing is performed using the following linear model

$$y=F\alpha +g(X,A)\;\text{β}+\psi ,\;\mathrm{with}\;\psi ∼N(0,\;\sigma_{n}^{2}I_{N}),$$

where *y* is the phenotype vector (*N* × 1) for *N* individuals, *F* the covariate matrix (*N* × *K*) for *K* covariates, and *α* the vector of covariate effects (*K* × 1). The function *g*(*X*, *A*) computes a gene-level burden score (*N* × 1), by aggregating the variant matrix *X* (*N* × *S*), where *S* is the number of rare variants, using the annotation matrix *A* (*S* × *L*), where *L* is the number of annotations per variant. The coefficient *β* represents the effect of the burden test, while *ψ* is the residual error, assumed to follow a normal distribution with variance $\sigma_{n}^{2}$. A classical choice for *g*(*X*, *A*) is the sum of pLoF variants within a gene for each individual (Cirulli et al. 2020). Within this framework, statistical association between the gene burden score and the phenotype is assessed by testing whether *β* ≠ 0.

#### Bayesian formulation

In BayesRVAT, we parameterize the aggregation function *g*_*φ*_(*X*, *A*) with parameters *ϕ* and introduce a prior distribution *p*(*ϕ*) that incorporates our prior beliefs on how to aggregate variants into a burden score based on their annotations (Fig. 1A):

$$y=F\alpha +g_{\phi }(X,A)\text{β}+\psi .$$

Introducing compact notations for input data *D* = {*F*, *X*, *A*} and model parameters *θ* = {*α*, *β*, *σ*^2^}, the model marginal likelihood can be written as

$$p(y|\;D,\theta )\;=\int p(y|\;D,\theta )p(\phi )d\phi \;=\;\int N(y\;|\;F\alpha +g_{\phi }(X,A)\text{β},\sigma^{2}I)p(\phi )d\phi .$$

We note that our Bayesian framework allows the data for each gene and trait to update these prior beliefs, effectively adapting the posterior on aggregation parameters *p*(*ϕ*) to the specific gene/trait pair being analyzed.

#### Choice of aggregation function and priors

After preprocessing annotations *A* to ensure that higher values correspond to more deleterious effects, we assume linear variant effects in *A* and use an additive model with saturation to collapse the contributions of multiple variants into a single gene burden score, *g*_*φ*_(*X*, *A*) = *σ* (*XAφ* − *b*_0_). Here, *b*_0_ is a bias term that ensures individuals carrying no rare variants receive a burden score close to zero, and the sigmoid function introduces a saturation mechanism—once the gene is impaired, additional variants no longer contribute to disruption. Our priors on *ϕ* reflect biological knowledge (Supplemental Fig. S1), setting pLoF effects’ prior such that carriers are highly likely to receive a gene burden score close to one. In contrast, for other nonsynonymous variants, we use weaker priors with greater variability, accounting for the uncertainty on their effects. For functional, regulatory, and splicing annotation scores, we apply priors that allow moderate, positive adjustments to the burden score (Supplemental Fig. S1).

#### Continuous and binary phenotypes

BayesRVAT supports both continuous and binary traits by adapting the phenotype likelihood function accordingly. For continuous traits, we assume a normal likelihood (as described above). For case-control traits, we use a Bernoulli likelihood with a sigmoid link function to map the linear predictor (including covariate and gene burden effects) to the probability of case status. Whereas all model derivations are presented in the continuous trait case, the adaptation to binary traits follows directly by substituting the likelihood function.

#### Optimization

The optimization of model parameters *θ* by maximum likelihood is intractable for a general aggregation function *g*_*φ*_(*X*, *A*) due to the integral over *ϕ*. We thus use black-box variational inference (Ranganath et al. 2014), which approximates the true posterior *p*(*ϕ* |*y*, *D*, *θ*) with a simpler variational distribution *q*_*ψ*_ (*φ*) parameterized by *ψ*. Within this framework, we optimize both the model parameters *θ* and the variational parameters *ψ* by maximizing the evidence lower bound (ELBO)

$$ELBO(\theta ,\psi )\;=E_{q_{\psi }(\phi )}\left[\log\;p(y\;|D,\theta ,\phi )\;-\log\left(\frac{q_{\psi }(\phi )}{\;p(\phi )}\right)\right].$$

We assume a mean-field Gaussian variational posterior for *q*_*ψ*_ (*φ*) and approximate the expectation using Monte Carlo sampling (Kingma and Welling 2014; Rezende et al. 2014). By maximizing the ELBO, we jointly estimate the values of $\hat{\theta }$ and the variational parameters $\hat{\phi }$, providing approximations of the maximum likelihood estimator of *θ* and the exact posterior distribution, respectively. To assess the accuracy of this approximation, we compared aggregation posteriors estimated through BayesRVAT with Markov chain Monte Carlo (MCMC) inference using Stan (Carpenter et al. 2017) on simulated phenotypes, finding that posterior means were consistent across methods, with narrower uncertainty estimates from BayesRVAT (Supplemental Fig. S14).

#### Association testing

Within the BayesRVAT framework, we can assess associations between gene burden scores and trait values by testing the hypothesis *β* ≠ 0 (Fig. 1B). As the likelihood ratio test statistic is intractable due to the integral over *ϕ*, in the alternative hypothesis, we introduce an approximate likelihood ratio test statistic. Briefly, we replace the intractable log marginal likelihood under the alternative hypothesis with the importance-weighted variational evidence lower bound (IW-ELBO) (Burda et al. 2015)

$$IW\text{-}ELBO(\hat{\theta },\hat{\psi })=E_{\phi_{1},\ldots \;,\phi_{K}∼\;q_{\hat{\psi }}(\phi )}\left[\log\left(\frac{1}{K}\Sigma_{k=1}^{K}\frac{\;p(y|\;D,\;\hat{\theta },\;\phi_{k})p(\phi_{k})}{\;q_{\hat{\psi }}(\phi_{k})}\right)\right],$$

which is computed using the approximate maximum likelihood estimators $\hat{\theta }$ and the variational posterior $\;q_{\hat{\psi }}(\phi )$, obtained through optimizing the ELBO. The IW-ELBO is a tighter bound on the log marginal likelihood compared to the standard ELBO, and its tightness increases with the number of importance samples *K*. Because the log marginal likelihood under the alternative hypothesis is replaced by its lower bound, whereas the null likelihood is computed exactly, the resulting test statistics are lower than those from an exact likelihood ratio test, yielding correspondingly higher *P*-values. Increasing the number of importance samples improves the approximation but entails higher computational cost (Supplemental Fig. S15).

#### Annotation importance scores

Similar to sensitivity analysis (Iooss and Lemaître 2015), we can evaluate the importance of a set of annotations by comparing the gene burden scores computed using all annotations (denoted as *A*_1_) with the scores obtained by setting a subset of annotations to their median values (denoted as *A*_0_). Specifically, we compute the expected value of the difference between these two burden scores

$$s\;=E_{\phi ∼q_{\hat{\psi }}(\phi )}[g_{\phi }(X,A_{1})-g_{\phi }(X,A_{0})].$$

The result *s* ∈ *R*^*N*×1^ quantifies how much each individual's gene burden is influenced by the annotations under investigation. We refer to these scores as annotation importance scores (Fig. 1C).

#### Implementation details

We implemented BayesRVAT and its derivatives using Numpy and SciPy, optimizing the ELBO using the L-BFGS in *scipy.optimize* (Byrd et al. 1995; Zhu et al. 1997; Morales and Nocedal 2011). Because L-BFGS requires noise-free loss and gradient evaluations, we fixed the Monte Carlo samples to approximate the expectation in the ELBO during its optimization (Loh et al. 2015). We empirically found 16 Monte Carlo samples to be sufficient in practice, as repeating the UKB blood trait analysis with independent draws yielded concordant results in the blood test analysis (Supplemental Fig. S16). After optimization, association *P*-values were computed by approximating the IW-ELBO using 30 Monte Carlo estimates, each based on 16 importance samples. We empirically validated the choice of 16 importance samples, observing nearly identical rankings when compared with larger *K* (Supplemental Fig. S17).

### Preprocessing of the UK BioBank data set

All experiments were conducted using the UKB cohort (Bycroft et al. 2018) based on the latest whole-exome sequencing (WES) release. Individual and variant quality control (QC) followed the protocols from Genebass (Karczewski et al. 2022). Variant annotation was performed using the pipeline described in Clarke et al. (2024). The final processed data set includes 329,087 unrelated European individuals, 5,845,828 variants with MAF < 0.1%, 16,458 genes, and 25 variant annotations.

#### Genetic data QC

We closely followed Karczewski et al. (2022) for variant QC. For sample QC, we followed the procedure used by the Neale Lab resource at https://www.nealelab.is/uk-biobank/. Briefly, we applied filters to remove samples flagged as used in PCA calculations (i.e., unrelated samples) and those with sex chromosome aneuploidy. To restrict the data set to individuals of British ancestry, we utilized the provided principal components (PCs), selecting individuals within seven standard deviations from the first six PCs, and filtered to self-reported ethnicities, specifically “white-British,” “Irish,” or “White.” To account for batch effects in whole-exome sequencing, we followed a similar approach to Karczewski et al. (2022). Briefly, we assessed the coverage around genes and used SCANPY (Wolf et al. 2018) to cluster individuals based on eight PCs of the coverage (10 nearest neighbors, Leiden clustering with resolution of 1). Smaller outlying clusters were excluded, and WES batch clusters were inferred using the Leiden clustering at a lower resolution (resolution 0.1), identifying three main groups. These cluster labels were used as covariates in downstream association studies to mitigate any potential batch-related bias (Law et al. 2014; Li et al. 2023).

#### Variant annotation

We defined consequences using VEP (McLaren et al. 2016) and classified pLoF as any splice donor, frameshift, splice acceptor, stop-gained, stop-lost, or start-lost variant. Our total set of 25 annotations consists of three consequence-based annotations consequences (pLoF, missense, and others), minor allele frequency, CADD (Rentzsch et al. 2019), SIFT (Kumar et al. 2009), PolyPhen-2 (Adzhubei et al. 2010), PrimateAI (Sundaram et al. 2018), Condel (González-Pérez and López-Bigas 2011), SpliceAI delta score (Jaganathan et al. 2019) and the AbSpliceDNA score (Wagner et al. 2023), eight RNA-binding protein binding propensity delta scores derived from DeepRiPE (Ghanbari and Ohler 2020), and six regulatory scores, derived as principal components of DeepSEA delta embeddings (Zhou and Troyanskaya 2015). We used the Phred scale to ensure consistency across annotations, that is, −10log_10_(rank(−score)/*L*), where *L* is the total number of variants (Li et al. 2020), with all annotations oriented so that higher values correspond to higher pathogenicity (e.g., by reversing SIFT, MAF, etc.).

### Benchmarking against alternative burden testing methods

To assess the performance of BayesRVAT compared to other commonly used burden testing techniques, we benchmarked our method against: pLoF-Burden, a burden test based on the sum of pLoF variants; ACAT-Conseq, which performs separate association tests on the sums of pLoF, missense, and other nonsynonymous variants, with *P*-values aggregated using the aggregated Cauchy association test (Liu et al. 2019)—a strategy similar to the simple allelic series models considered in McCaw et al. (2023); and ACAT-MultiAnnot, which runs burden tests across each of the annotations used in BayesRVAT, combining results using ACAT, similar to the approach in Li et al. (2020). All burden tests were implemented as likelihood ratio tests within a linear model framework, adjusting for age, sex, the top 20 genetic principal components, and WES batch effect covariates. For continuous traits (Gaussian likelihood), maximum likelihood estimates have a closed-form solution, whereas for binary traits (Bernoulli likelihood), we optimize the null and alternative models using L-BFGS, implemented in SciPy.

### Simulations

We used synthetic data to assess both the calibration and power of BayesRVAT under various simulated conditions, based on 100,000 individuals from the processed UKB cohort. To evaluate power, we simulated additive genetic effects from a gene burden test using the additive model with saturation assumed in BayesRVAT. We varied key parameters such as sample size, variance explained by the burden, and the number of continuous annotations contributing to the burden score. Power was estimated at the exome-wide significance threshold of *P* < 2.5 × 10^−6^, with 100 replicates performed for each simulation configuration. We additionally evaluated robustness by introducing annotation-independent random effects, creating scenarios where causal architectures deviated from the BayesRVAT allelic series assumptions. We also assessed power and calibration for binary traits with different prevalences. Finally, to assess calibration, we simulated phenotypes under a null model with no genetic effects. Full details on the simulation framework are provided in Supplemental Material.

### Real data analyses in UK Biobank

#### Blood biomarkers

We applied BayesRVAT and baselines to exome sequencing data from the UK Biobank, selecting unrelated individuals of self-reported White British ancestry. We analyzed 12 key blood biomarkers: LDL cholesterol, HDL cholesterol, apolipoprotein A, apolipoprotein B, triglycerides, glycated hemoglobin, alanine aminotransferase, aspartate aminotransferase, albumin, alkaline phosphatase, calcium, and C-reactive protein. To ensure normality, all biomarker phenotypes were transformed using a rank-based inverse normal transformation.

#### Integration with SKAT

We used the classical SKAT variance component test and combined its results with each burden test using the aggregated Cauchy association test (ACAT), forming an optimal test. Note that, in comparison with SKAT-O (Supplemental Figs. S4, S9), we used the standard SKAT-O implementation in R (Lee et al. 2012; R Core Team 2023) rather than this ACAT-based equivalent.

#### Disease code analyses

We analyzed eight disease traits: type 2 diabetes (*fieldID* = 130708; 26,328 cases, 302,759 controls), asthma (*fieldID* = 131494; 47,125 cases, 281,962 controls), coronary artery disease (*fieldID* = 131306; 33,878 cases, 295,209 controls), age-related macular degeneration (*fieldID* = 131182; 11,083 cases, 318,004 controls), glaucoma (*fieldID* = 131186; 12,633 cases, 316,454 controls), cataract (*fieldID* = 131166; 30,566 cases, 298,521 controls), atrial fibrillation (*fieldID* = 131350; 25,813 cases, 303,274 controls), and hypertension (*fieldID*=131286; 126,992 cases, 202,095 controls). These conditions are relatively common (≥5000 cases in UK Biobank) and span respiratory, metabolic, cardiovascular, and ocular diseases. All traits were derived from “Date first reported” phenotypes, curated by UK Biobank using ICD codes, primary care records, and self-reported data. For each trait, we tested 16,017 genes, yielding a total of 128,136 gene–trait association tests. Statistical significance was defined using a Bonferroni-adjusted *P* < 0.05 (raw *P* < 3.9 × 10^−7^).

### Computational complexity and run time

BayesRVAT is optimized for biobank-scale analyses and exhibits linear computational complexity with respect to sample size. To further improve efficiency, we exploit that most individuals in rare variant studies carry no variant in a given gene—for these, the ELBO expectation collapses to the closed-form null likelihood, eliminating Monte Carlo evaluations for the majority of individuals. We empirically assessed run time scaling by measuring the average time to fit a single gene at different cohort sizes in simulations (Supplemental Fig. S13). With the sparse implementation, run times were 0.23 ± 0.06 sec for N = 50,000, 0.36 ± 0.11 sec for N = 100,000, 1.2 ± 0.41 sec for N = 200,000, and 1.65 ± 0.58 sec for N = 300,000 individuals. To further reduce computational burden, we propose a two-stage filtering strategy, applying BayesRVAT selectively to genes showing preliminary association signals in simpler burden tests. Because most genes are not associated with the phenotype and yield approximately uniform *P*-values under the null, applying a prefiltering threshold of *P* < 10^−2^ reduces the number of genes analyzed by 90%, cutting total run time to <∼47 min for N = 300,000. All run times were estimated on Intel Xeon Gold 6134 CPUs with 32 logical cores.

### Use of artificial intelligence

In the preparation of this manuscript, we utilized the large language model GPT-4 (https://chat.openai.com/) for editing assistance, including language polishing and clarification of text. Although this tool assisted in refining the manuscript's language, it was not used to generate contributions to the original research, data analysis, or interpretation of results. All final content decisions and responsibilities rest with the authors.

### Software availability

An open-source software implementation of BayesRVAT is available at GitHub (https://github.com/AIH-SGML/BayesRVAT) and as Supplemental Code.

## Supplemental Material

Supplement 1

Supplement 2

Supplement 3
