# Supplementary material for: BayesRVAT enhances rare-variant association testing through Bayesian aggregation of functional annotations
Source: Genome Res. 2025 Dec;35(12):2682–90. doi: 10.1101/gr.280689.125 (PMC12667389; doi:10.1101/gr.280689.125)
Supplement: Supplement 1 [file Supplemental_Material.pdf]

Supplemental Material for "BayesRVAT enhances rare-variant association testing through Bayesian aggregation of functional annotations"

Contents

A1 Supplemental Information **1**
A1.1 Related work . . . 1
A1.2 Simulation design . . . 2

A2 Supplemental Figures **4**

A3 Supplemental Tables **21**

A1 Supplemental Information

A1.1 Related work

Burden tests. While our Bayesian RVAT framework is general, we here specialize it to perform burden tests. Burden tests aggregate the effects of rare variants into a gene burden score to improve statistical power. This score is then regressed against a trait of interest in a formal test [4, 14, 13, 19, 23, 20]. While traditional burden tests have been limited to few annotations such as minor allele frequency (MAF) and variant consequences [8, 14], BayesRVAT can integrate multiple annotations leveraging a Bayesian framework.

Allelic series. BayesRVAT can model gene-level effects as a function of multiple rare variants and their functional annotations, effectively capturing allelic series. The term "allelic series" refers to a collection of variants within a gene that exhibit a gradation of phenotypic effects based on their severity, suggesting a dose-response relationship between gene functionality and the resulting phenotype [22, 21]. As allelic series

enable the assessment of the feasibility of pharmacological modulation [21, 6, 17], methods that can accurately capture these relationships are of significant interest. For example, COAST models allelic series by weighting variants based on the expected deleteriousness of few functional consequences [17]. Conversely, DeepRVAT uses a data-driven approach to learn a trait-gene-agnostic aggregation function from multiple annotations using neural networks [5]. In contrast to these methods, BayesRVAT can handle larger sets of variant annotations without enforcing a universally fixed scheme across genes and traits.

Variance component models. In contrast to burden tests, which assume a uniform effect direction across all variants, variance component approaches allow for both deleterious and protective effects by employing random effect models. The most widely used variance component test for rare variants is SKAT [28]. Given the complementary strengths of burden and variance component tests [1], omnibus tests that combine both, such as SKAT-O [12], have become increasingly popular[18, 15, 32]. In this work, we demonstrate that BayesRVAT integrates smoothly within omnibus test procedures, maintaining its power advantages over other integrated burden tests.

Bayesian inference. BayesRVAT performs Bayesian inference on parameters modeling variant effects as a function of multiple annotations. Given the intractability of exact posterior computation, we use black-box variational inference [24], which reformulates the inference problem as an optimization task, directly optimizing a variational distribution to approximate the true posterior using gradient-based methods [2, 25, 11, 7, 26]. While Bayesian methods for RVAT have been previously explored [30, 27, 16, 31], BayesRVAT is the first unified Bayesian framework that can incorporate multiple genetic architectures and variant annotations.

A1.2 Simulation design

Briefly, for each random seed, we simulated the phenotype as follows:

- For null simulations, no genetic effects were included and phenotypes were sampled as standard normal noise across individuals;
- For alternative simulations, we randomly selected C contributing continuous annotations;
- The gene burden was built using the additive model with saturation

$$g_{\boldsymbol{\phi}}(\boldsymbol{X}, \boldsymbol{A}) = \text{sigmoid}(\boldsymbol{X}(\boldsymbol{A}\boldsymbol{\phi}) - b_0). \tag{A.1}$$

  where the effects of pLoF and missense were sampled from their respective priors, and the effects of the C selected contributing continuous annotations were sampled from $\mathcal{N}(4, 0.01)^*$;
- To evaluate robustness under model mismatch, we simulated the total genetic effect as

$$\boldsymbol{g} = \sqrt{1-\eta}\,\boldsymbol{b} + \sqrt{\eta}\,\boldsymbol{u},$$

*This ensures that the selected contributing annotations have a measurable effect on the burden score, thus effectively controlling the number of contributing continuous annotations C.

where $\boldsymbol{b}$ is the burden and $\boldsymbol{u}$ is the standardized $\boldsymbol{X}^{\star}\boldsymbol{\beta}$. The matrix $\boldsymbol{X}^{\star}$ was obtained from $\boldsymbol{X}$ by (i) collapsing ultra-rare variants (MAC$<$ 10) and (ii) applying SKAT-style MAF-based weights $w_j = \sqrt{\text{Beta}(\text{maf}_j; 1, 25)}$ [28], with $\boldsymbol{\beta} \sim \mathcal{N}(0, \boldsymbol{I})$. The parameter η controls the degree of mismatch, with $\eta = 0$ corresponding to a pure burden model.

- The burden was standardized to have a mean of 0 and standard deviation of 1 across individuals;
- The phenotype $\boldsymbol{y} \in \mathbb{R}^{N\times 1}$ was simulated as:

$$\boldsymbol{y} = \sqrt{v_g} \cdot \boldsymbol{g} + \sqrt{1 - v_g} \cdot \boldsymbol{\psi}, \quad \boldsymbol{\psi} \overset{\text{iid}}{\sim} \mathcal{N}(0, 1), \tag{A.2}$$

  where $\boldsymbol{g} \in \mathbb{R}^{N\times 1}$ is the standardized total genetic effects and v_g represents the corresponding proportion of explained variance.
- For binary traits, we applied a threshold to the simulated continuous phenotype to generate case–control labels. To ensure comparable power with the continuous case for benchmarking, we increased the default value of the total genetic variance from 3×10^{-4} to 5×10^{-4}.

In this framework, we evaluated power of BayesRVAT and other burden tests varying C, v_g, and the sample size.

A2 Supplemental Figures

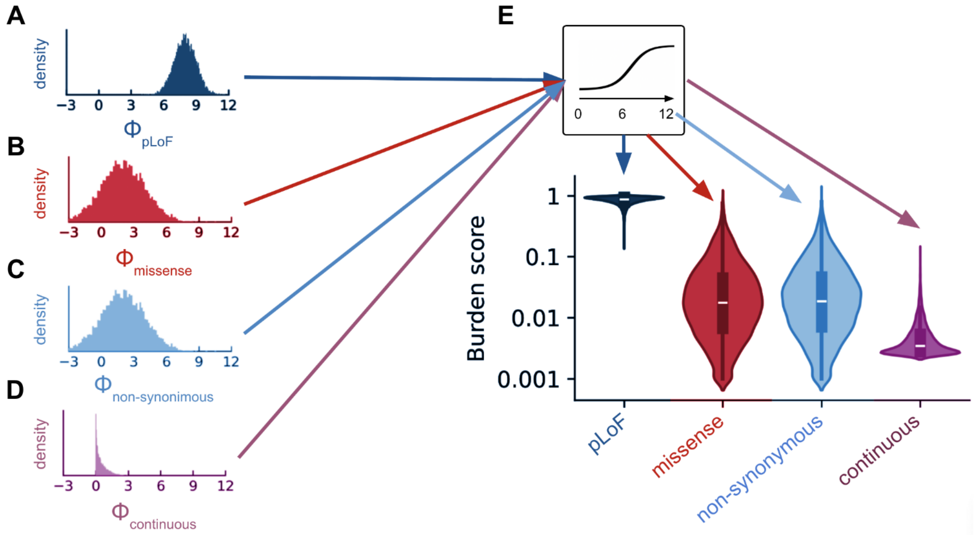

Supplemental Figure S1 | Prior distributions for variant annotations used in BayesRVAT. (**A**–**D**) Prior distributions over the raw annotation weights $\boldsymbol{\phi}$, before applying the shift b_0 and the sigmoid transformation in the aggregation function $g_{\boldsymbol{\phi}}(X, A) = \sigma(\boldsymbol{X}\boldsymbol{A}\boldsymbol{\phi} - b_0\mathbf{1}_{N\times 1})$. (**A**) pLoF variants are modeled with a normal prior (mean = 8, standard deviation = 1). (**B**) Missense variants are modeled with a normal prior (mean = 2, standard deviation = 2). (**C**) Other non-synonymous variants follow a normal prior (mean = 2, standard deviation = 2). (**D**) Continuous annotations, including regulatory and functional scores, are modeled with softplus-transformed Gaussian priors (mean = 0, standard deviation = 1, β = 2), ensuring positive contributions to the burden score. (**E**) Translation of annotation-level priors into distributions of gene burden scores. pLoF variants, reflecting their strong and disruptive biological impact, are highly likely to yield burden scores near one. Missense and other non-synonymous variants contribute more weakly and variably, while continuous regulatory scores, which are often noisier and gene- or trait-specific, contribute only small positive effects. Together, this illustrates how BayesRVAT can be viewed as a pLoF-based test that flexibly incorporates additional annotations, consistent with long-standing biological knowledge.

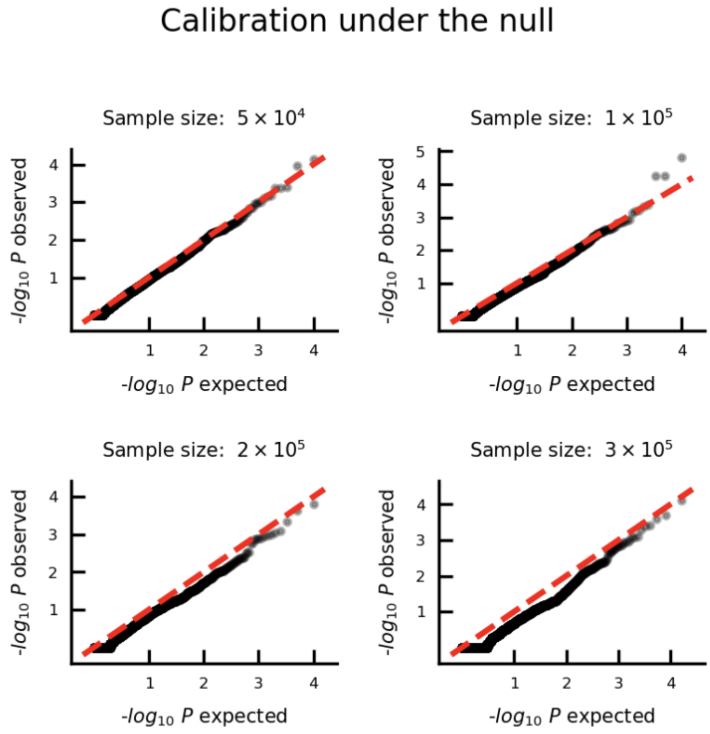

Supplemental Figure S2 | Assessment of calibration of BayesRVAT in simulations varying sample size. Shown are QQ plots of P values from BayesRVAT under a null model with no genetic effects, using simulated phenotypes for 50,000, 100,000, 200,000, and 300,000 unrelated individuals from the UK Biobank (**Methods**).

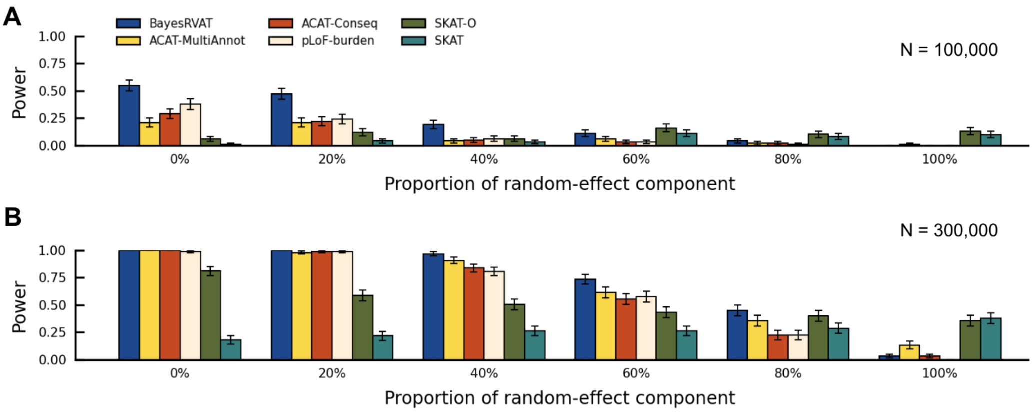

Supplemental Figure S3 | Power under model mismatch with annotation-independent random effects. We simulated causal architectures where variant effects included annotation-independent random-effect components not captured by the modeled annotation set, creating increasing mismatch between the true effects and BayesRVAT's allelic series prior. Results are shown for N = 100,000 (**A**) and N = 300,000 (**B**). As the contribution of the random-effect component increased, all methods lost power. BayesRVAT retained a clear advantage over alternative burden tests across most configurations, while variance-component methods such as SKAT-O outperformed BayesRVAT when the signal was dominated by random effects. Error bars represent standard errors across simulation replicates.

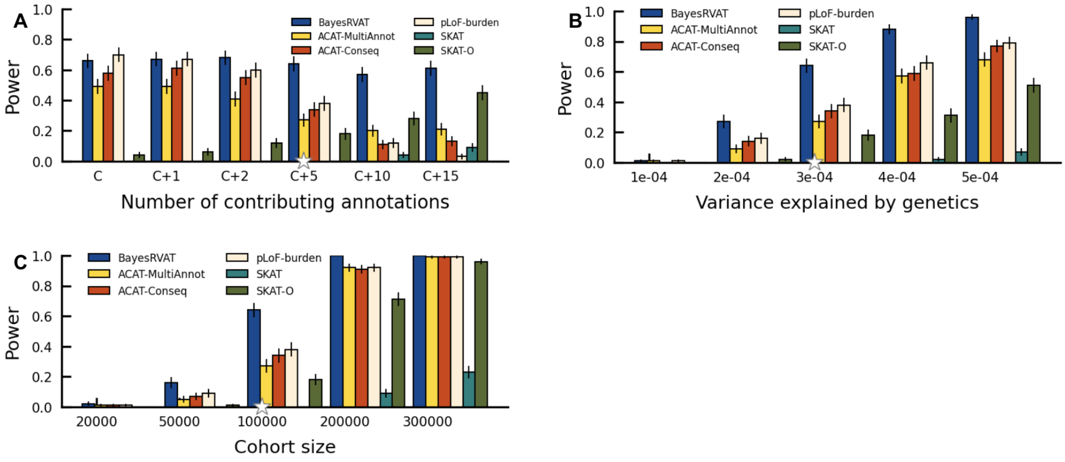

Supplemental Figure S4 | Power assessment of BayesRVAT across different contributing annotations to the phenotype, cohort sizes and variance explained by genetic effects. (**A-C**) Statistical power of BayesRVAT compared to pLoF-burden, ACAT-Conseq, ACAT-MultiAnnot, SKAT, and SKAT-O across varying number of contributing annotation to the phenotype (**A**) variance explained by genetics (**B**) and cohort size (**C**). Power is measured at the exome-wide significance threshold of $P < 2.5 \times 10^{-6}$, with results averaged over 100 random seeds for each setting.

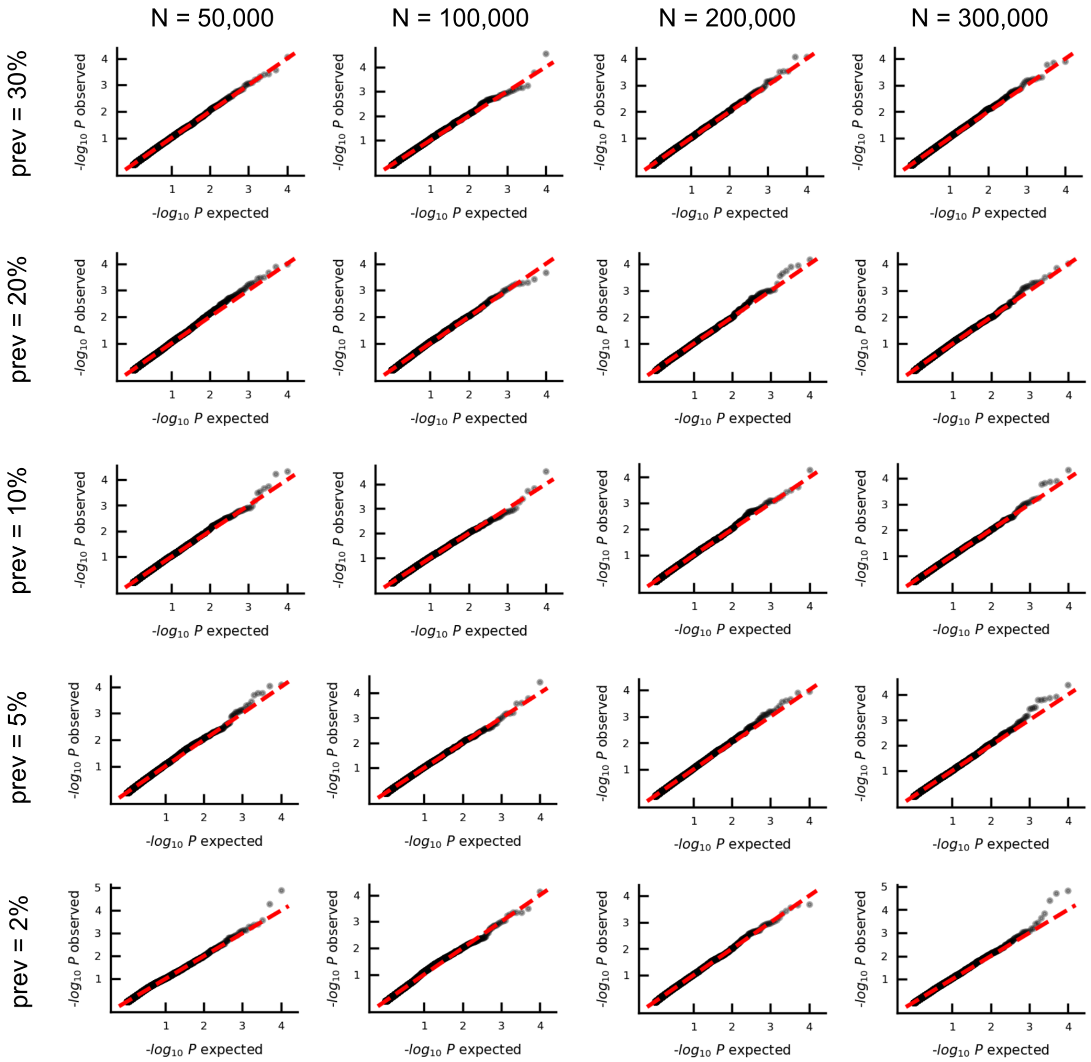

Supplemental Figure S5 | Calibration of BayesRVAT for binary phenotypes. Quantile–quantile (QQ) plots of observed versus expected P-values under the null for case–control traits across different sample sizes (N = 50,000 to N = 300,000) and prevalences (30% to 2%). In all settings, BayesRVAT maintains well-calibrated test statistics, even at low prevalence and large sample sizes. Red dashed lines indicate the expected null distribution. These results confirm that BayesRVAT retains valid calibration for binary traits across a broad range of realistic study designs.

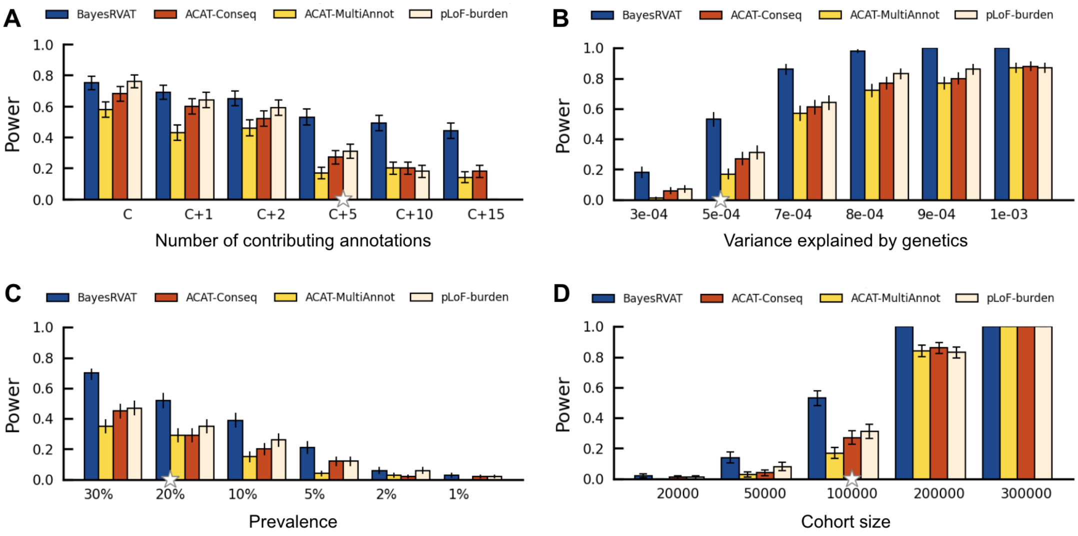

Supplemental Figure S6 | Power of BayesRVAT for binary phenotypes compared to alternative burden tests. Power was evaluated across a range of simulation scenarios and compared with ACAT-Conseq, ACAT-MultiAnnot, and pLoF-burden. (**A**) Increasing number of contributing annotations. (**B**) Increasing proportion of variance explained by genetics. (**C**) Varying case–control prevalence. (**D**) Increasing cohort size. Across all conditions, BayesRVAT demonstrates higher power, particularly when multiple annotations contribute modest effects or when sample size and trait prevalence are limited. Error bars indicate standard errors across simulation replicates.

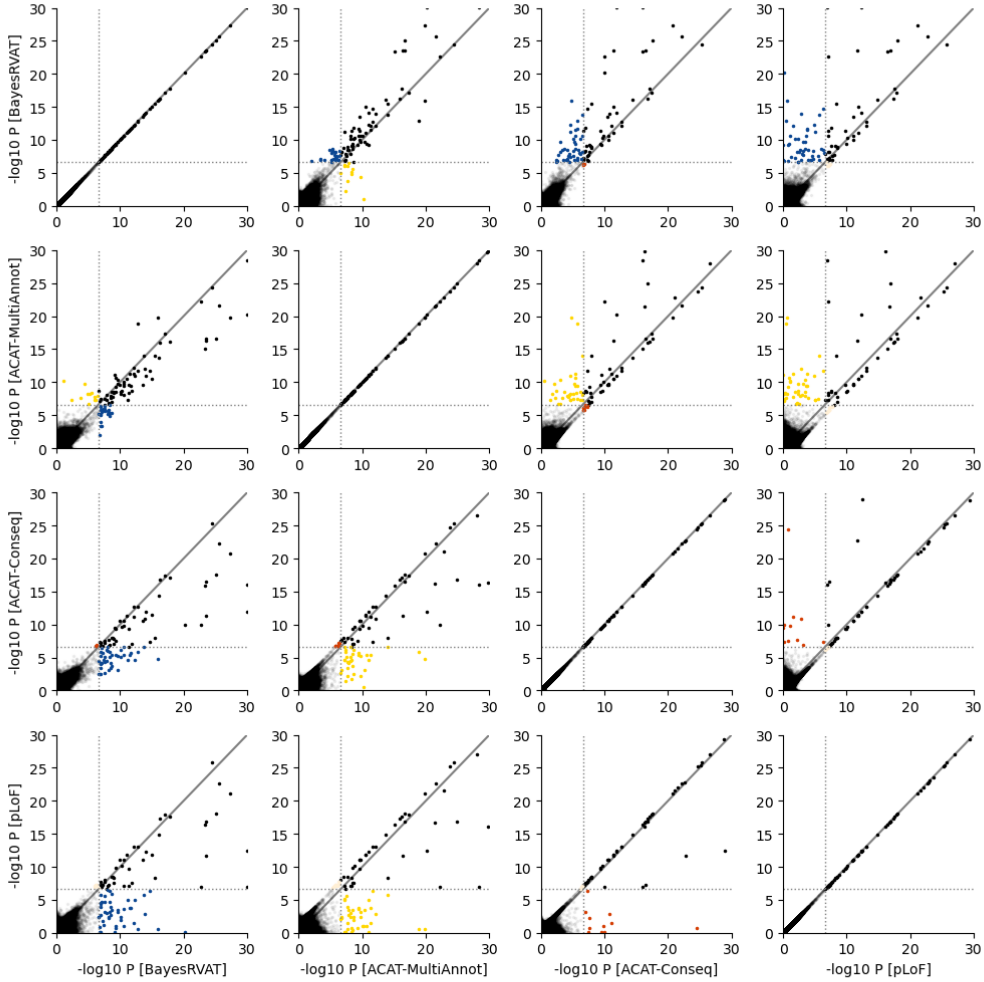

Supplemental Figure S7 | Comparison of P-values from different burden test strategies in the blood trait analysis. Each scatter plot compares the $-log_{10}$ P-values from two different methods, where points represent individual gene-trait pairs. Diagonal lines indicate equal P-values for the two methods being compared. Blue, yellow, red and pink points highlight gene-trait pairs uniquely identified by BayesRVAT, ACAT-MultiAnnot, and ACAT-Conseq, and pLoF, respectively, that are not detected by the other method. BayesRVAT recapitulates most signals from other tests, recovering additional associations beyond consequence-based burden tests and boosting nominal signals relative to ACAT-MultiAnnot in allelic series settings.

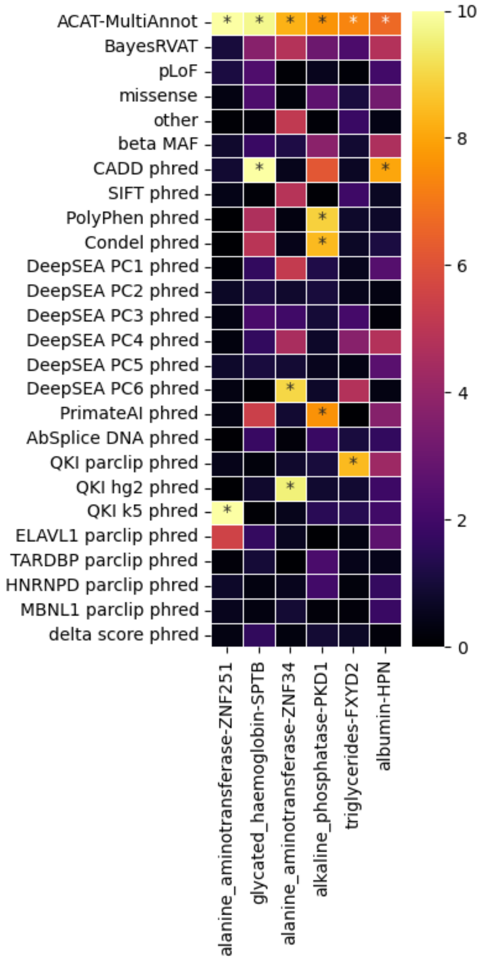

Supplemental Figure S8 | Violations of the allelic series assumptions in BayesRVAT explain differences with ACAT-MultiAnnot. The heatmap displays $-log_{10}$ P-values for gene-trait associations across individual annotations tested in ACAT-MultiAnnot, as well as the overall results for BayesRVAT and ACAT-MultiAnnot. Each column corresponds to a gene-trait pair identified by ACAT-MultiAnnot but missed by BayesRVAT, and each row represents a different annotation or burden test. In all these cases, the loss of power for BayesRVAT can be explained by the violation of the allelic series assumption encoded in its prior—namely, that pLoF variants have stronger effects than other annotations. In these gene-trait pairs, certain annotations (e.g., CADD, PolyPhen, Condel) exhibit much stronger effects than pLoF, driving the associations in ACAT-MultiAnnot. Stars (*) denote annotations with P-values that pass the Bonferroni-adjusted significance threshold of $\alpha < 0.05$.

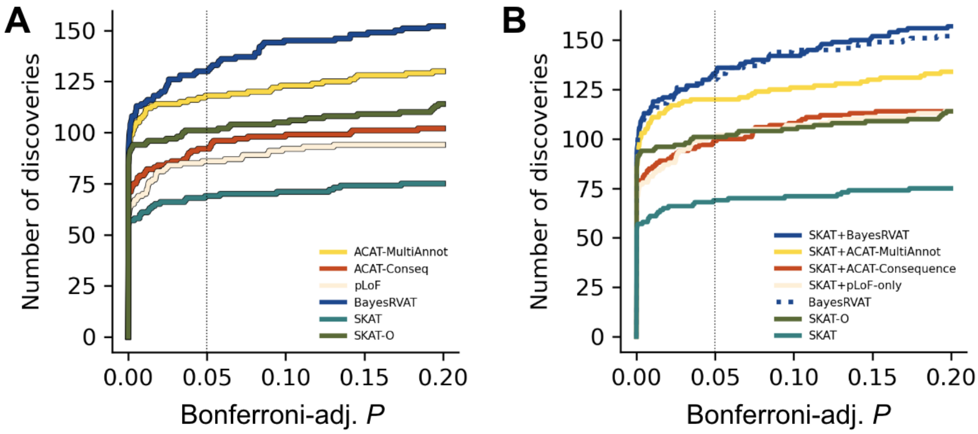

Supplemental Figure S9 | Analysis of blood biomarkers in the UK Biobank including SKAT and SKAT-O. (**A**) BayesRVAT outperform both burden (pLoF-Burden, ACAT-Conseq, and ACAT-MultiAnnot), variance components (SKAT) and optimal (SKAT-O) tests in number of discoveries at varying Bonferroni-adjusted significance thresholds α. (**B**) BayesRVAT shows a superior number of discoveries even when other methods are integrated with SKAT results.

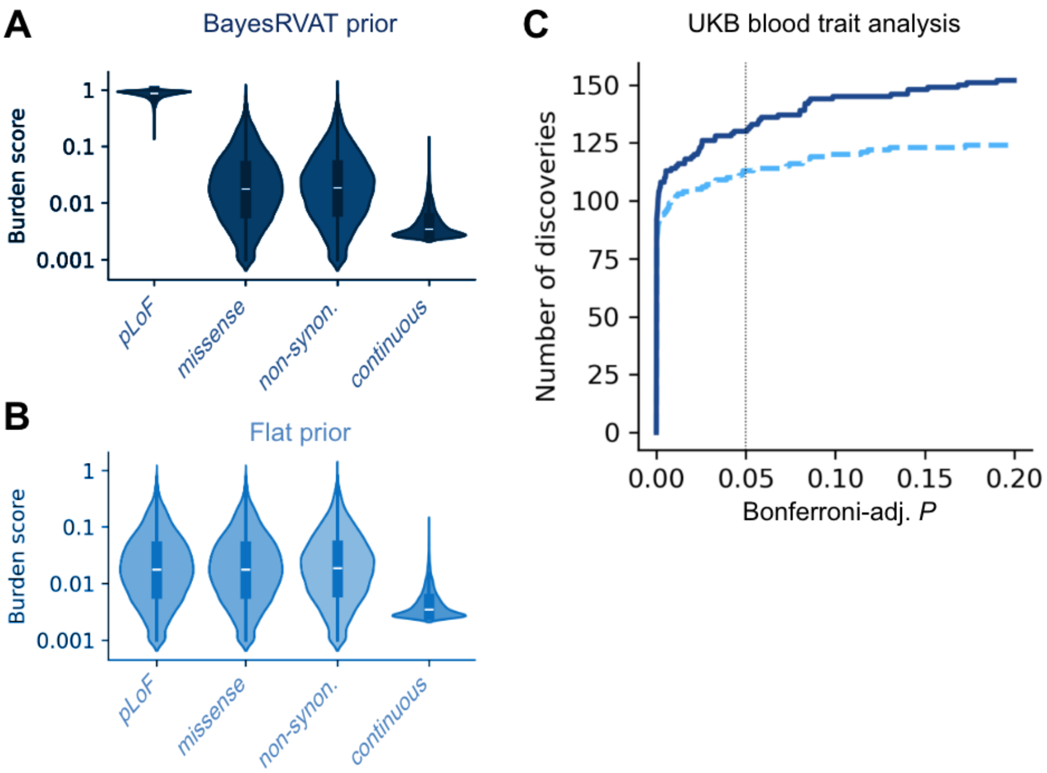

Supplemental Figure S10 | Comparison of biologically informed versus flat priors in BayesRVAT. (**A**–**B**) Distribution of burden scores under the biologically informed pLoF-dominant prior (**A**) and a flat prior across consequence classes (**B**). The pLoF-dominant prior yields burden scores concentrated near one for pLoF carriers, while other annotations contribute more variably and weakly. (**C**) UK Biobank blood trait analyses comparing the number of discoveries across significance thresholds. The pLoF-dominant prior (solid line) produces substantially more discoveries than the flat prior (dashed line), confirming that upweighting pLoF is both biologically justified and empirically beneficial.

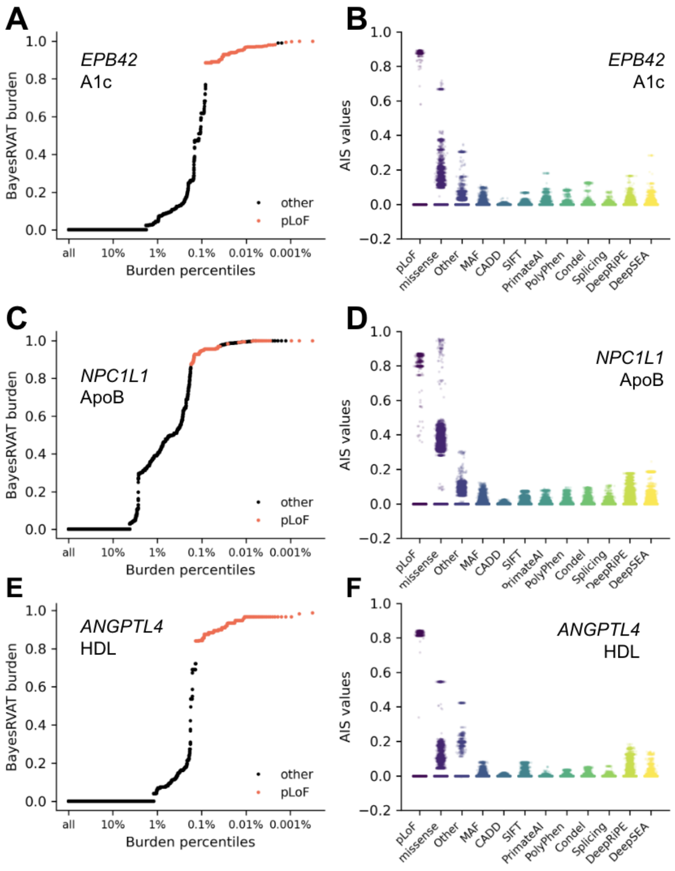

Supplemental Figure S11 | Burden score and annotation importance scores (AIS) for additional blood biomarker associations detected by BayesRVAT. (**A**) Burden scores learned by BayesRVAT for *EPB42* and HbA1c across burden percentiles, with individuals carrying pLoF mutations highlighted in red. This association aligns with recent findings showing a strong link between rare variants in *EPB42* and A1c levels [10]. (**B**) Annotation importance scores (AIS) for the association between *EPB42* and HbA1c, showing contributions from annotations such as missense, SIFT, and DeepSEA. (**C**) Burden scores for *NPC1L1* and apolipoprotein B (ApoB), with pLoF variants highlighted in red. *NPC1L1* is crucial for cholesterol absorption in the intestine and liver, and variants in this gene have been linked to altered ApoB levels, influencing lipid metabolism and cardiovascular disease risk [9]. (**D**) AIS for the association between *NPC1L1* and ApoB, highlighting the roles of missense and regulatory annotations. (**E**) Burden scores for *ANGPTL4* and HDL cholesterol, with pLoF variants highlighted in red. *ANGPTL4* is a key regulator of lipid metabolism, inhibiting lipoprotein lipase, which affects triglyceride breakdown and HDL cholesterol levels, with certain variants associated with increased HDL and cardiovascular protection[29]. (**F**) AIS for the association between *ANGPTL4* and HDL cholesterol, showing contribution from annotations such as missense, other non-synonymous, DeepRiPE, and DeepSEA.

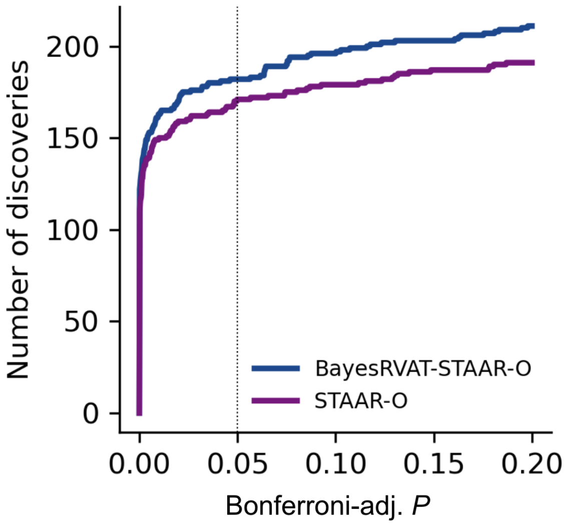

Supplemental Figure S12 | BayesRVAT improves gene–trait discovery when integrated into the STAAR omnibus framework in blood trait analysis. Cumulative number of significant gene–trait associations (y-axis) as a function of Bonferroni-adjusted P value thresholds (x-axis) for STAAR-O (purple) and BayesRVAT-STAAR-O (blue), which integrates BayesRVAT with all unit tests in STAAR. BayesRVAT-STAAR-O consistently yields more discoveries, indicating complementary signals captured by BayesRVAT.

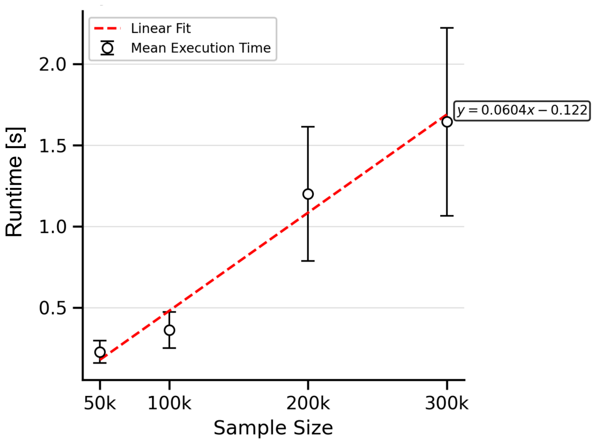

Supplemental Figure S13 | Runtime scalability of BayesRVAT. BayesRVAT scales linearly with sample size by design, enabling efficient application to large biobank datasets. Additional computational gains are achieved by exploiting the sparsity of rare variant burdens, allowing the ELBO expectation term to collapse to the exact log marginal likelihood for the 90–99% of individuals carrying no variant. Shown is the mean runtime as a function of sample size (50k–300k), with error bars denoting standard deviations across replicates and a linear fit (red).

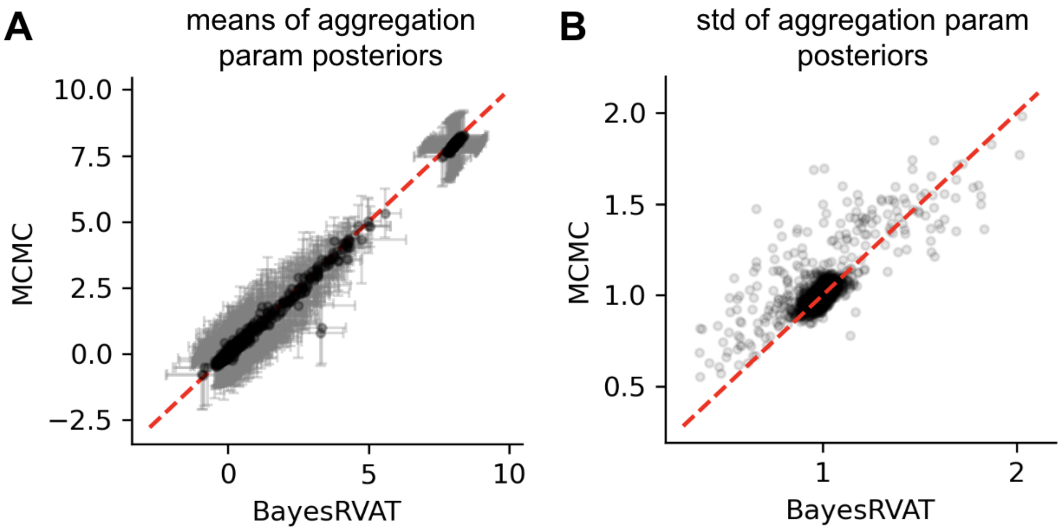

Supplemental Figure S14 | Comparison of BayesRVAT variational inference (VI) and full MCMC inference. We evaluated BayesRVAT's mean-field VI against Stan [3] on 100 simulated phenotypes at $N = 100{,}000$ under the default simulation design. (**A**) Posterior means of the aggregation parameters were highly consistent between VI and MCMC. (**B**) As expected, VI produced narrower posterior standard deviations than MCMC. These results confirm that BayesRVAT's VI approximation yields parameter estimates consistent with MCMC, while slightly underestimating posterior uncertainty.

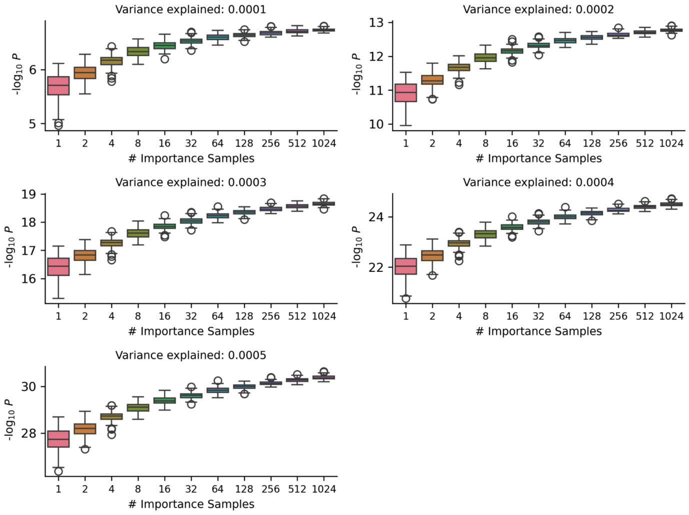

A Effect of varying K on test statistics.

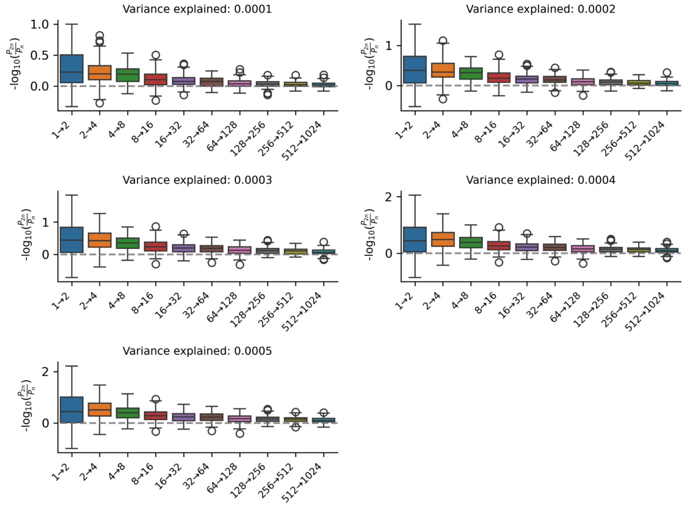

B Changes in P-values when doubling K.

Supplemental Figure S15 | Trade-off between computational efficiency and accuracy in the number of importance samples. We evaluated the effect of varying the number of importance samples (K) on BayesRVAT test statistics (**A**) and the corresponding changes in P-values when doubling K (**B**). Increasing K improves the accuracy of the importance-weighted approximation and yields slightly lower P-values, at the cost of higher computational demand. These results illustrate the trade-off between computational efficiency and precision, showing that performance stabilizes rapidly and that changes remain within one order of magnitude.

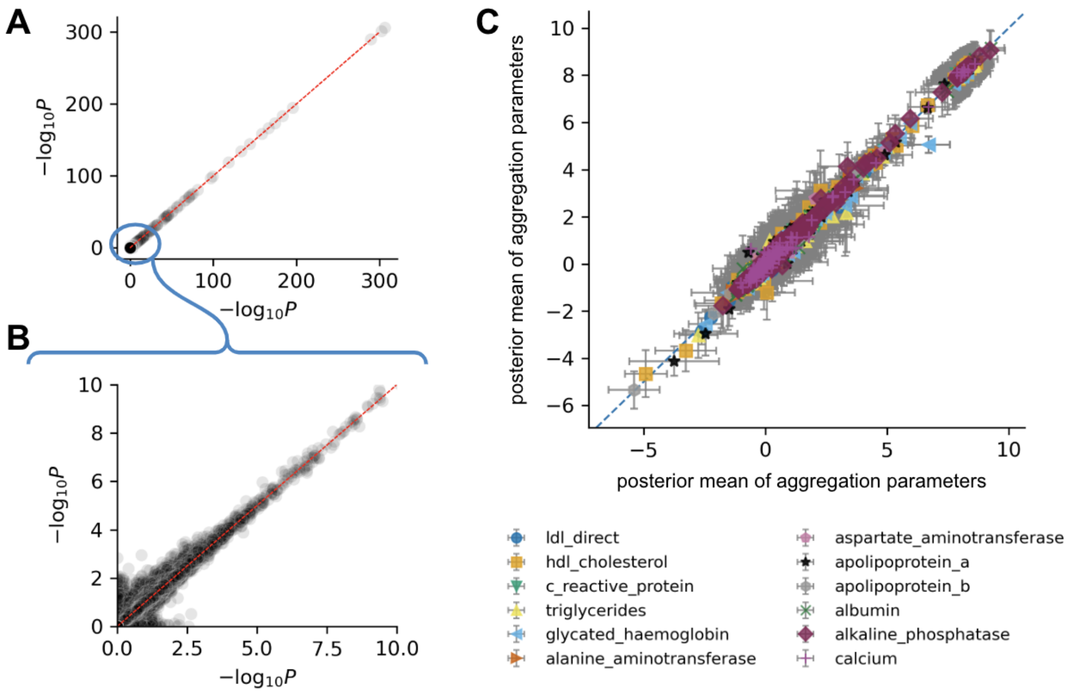

Supplemental Figure S16 | Robustness of BayesRVAT optimization to different Monte Carlo seeds. (**A**–**B**) Association P-values from two independent runs of the UK Biobank blood biomarker analysis, where each run used 16 fixed Monte Carlo samples but with different random seeds, resulting in distinct sets of samples. Results are shown across the full range (**A**) and at smaller scales (**B**). (**C**) Posterior means of the aggregation parameters from the two runs, with error bars indicating posterior standard deviations. The nearly identical outcomes confirm that BayesRVAT optimization yields consistent parameter estimates even when different fixed sets of Monte Carlo samples are used.

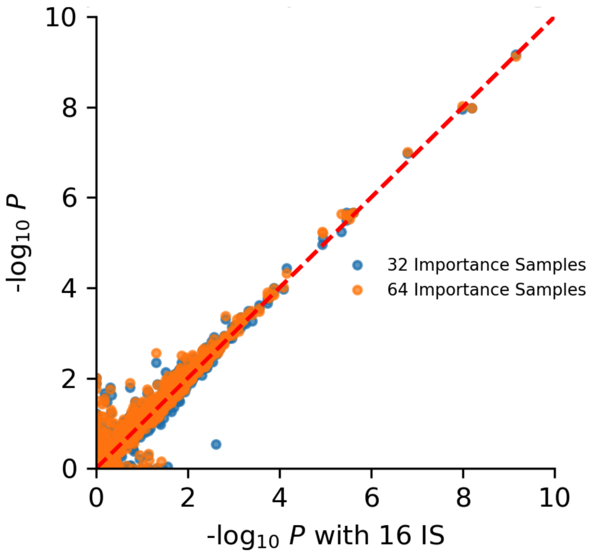

Supplemental Figure S17 | Empirical assessment of the choice of $K = 16$ importance samples (LDL analysis). Comparison of $-\log_{10} P$ values for LDL obtained with $K = 16$ (x-axis) versus $K = 32$ (blue) and $K = 64$ (orange). Results show nearly perfect correlations ($r \approx 0.98$), indicating that increasing K beyond 16 has minimal impact on hypothesis rankings. This analysis supports the use of $K = 16$ as a computationally efficient and empirically robust choice in BayesRVAT.

A3 Supplemental Tables

Supplemental Table S1 | Significant gene-trait associations from the analysis of eight disease traits. Shown are P values for pLoF-only (P_{pLoF}), ACAT-Consequence ($P_{\text{ACAT-Conseq}}$), ACAT-MultiAnnot ($P_{\text{ACAT-MultiAnnot}}$), and BayesRVAT ($P_{\text{BayesRVAT}}$), and effect size estimates (β) for pLoF and BayesRVAT burdens.

References

[1] Saonli Basu and Wei Pan. Comparison of statistical tests for disease association with rare variants. Genetic epidemiology, 35(7):606–619, 2011.

[2] David M Blei, Alp Kucukelbir, and Jon D McAuliffe. Variational inference: A review for statisticians. Journal of the American statistical Association, 112(518): 859–877, 2017.

[3] Bob Carpenter, Andrew Gelman, Matthew D Hoffman, Daniel Lee, Ben Goodrich, Michael Betancourt, Marcus Brubaker, Jiqiang Guo, Peter Li, and Allen Riddell. Stan: A probabilistic programming language. Journal of statistical software, 76: 1–32, 2017.

[4] Elizabeth T Cirulli, Simon White, Robert W Read, Gai Elhanan, William J Metcalf, Francisco Tanudjaja, Donna M Fath, Efren Sandoval, Magnus Isaksson, Karen A Schlauch, et al. Genome-wide rare variant analysis for thousands of phenotypes in over 70,000 exomes from two cohorts. Nature communications, 11 (1):542, 2020.

[5] Brian Clarke, Eva Holtkamp, Hakime Öztürk, Marcel Mück, Magnus Wahlberg, Kayla Meyer, Felix Munzlinger, Felix Brechtmann, Florian R Hölzlwimmer, Jonas Lindner, et al. Integration of variant annotations using deep set networks boosts rare variant association testing. Nature Genetics, pages 1–10, 2024.

[6] Calliope A Dendrou, Adrian Cortes, Lydia Shipman, Hayley G Evans, Kathrine E Attfield, Luke Jostins, Thomas Barber, Gurman Kaur, Subita Balaram Kuttikkatte, Oliver A Leach, et al. Resolving tyk2 locus genotype-to-phenotype differences in autoimmunity. Science translational medicine, 8(363):363ra149–363ra149, 2016.

[7] Jan P Engelmann, Alessandro Palma, Jakub M Tomczak, Fabian Theis, and Francesco Paolo Casale. Mixed models with multiple instance learning. In International Conference on Artificial Intelligence and Statistics, pages 3664–3672. PMLR, 2024.

[8] Fang Han and Wei Pan. A data-adaptive sum test for disease association with multiple common or rare variants. Human heredity, 70(1):42–54, 2010.

[9] Lin Jia, Jenna L Betters, and Liqing Yu. Niemann-pick c1-like 1 (npc1l1) protein in intestinal and hepatic cholesterol transport. Annual review of physiology, 73 (1):239–259, 2011.

[10] Young Jin Kim, Sanghoon Moon, Mi Yeong Hwang, Sohee Han, Hye-Mi Jang, Jinhwa Kong, Dong Mun Shin, Kyungheon Yoon, Sung Min Kim, Jong-Eun Lee, et al. The contribution of common and rare genetic variants to variation in metabolic traits in 288,137 east asians. Nature communications, 13(1):6642, 2022.

[11] Diederik P Kingma. Auto-encoding variational bayes. arXiv preprint arXiv:1312.6114, 2013.

[12] Seunggeun Lee, Michael C Wu, and Xihong Lin. Optimal tests for rare variant effects in sequencing association studies. Biostatistics, 13(4):762–775, 2012.

[13] Seunggeung Lee, Gonçalo R Abecasis, Michael Boehnke, and Xihong Lin. Rare-variant association analysis: study designs and statistical tests. The American Journal of Human Genetics, 95(1):5–23, 2014.

[14] Bingshan Li and Suzanne M Leal. Methods for detecting associations with rare variants for common diseases: application to analysis of sequence data. The American Journal of Human Genetics, 83(3):311–321, 2008.

[15] Xihao Li, Zilin Li, Hufeng Zhou, Sheila M Gaynor, Yaowu Liu, Han Chen, Ryan Sun, Rounak Dey, Donna K Arnett, Stella Aslibekyan, et al. Dynamic incorporation of multiple in silico functional annotations empowers rare variant association analysis of large whole-genome sequencing studies at scale. Nature genetics, 52 (9):969–983, 2020.

[16] Benjamin A Logsdon, James Y Dai, Paul L Auer, Jill M Johnsen, Santhi K Ganesh, Nicholas L Smith, James G Wilson, Russell P Tracy, Leslie A Lange, Shuo Jiao, et al. A variational bayes discrete mixture test for rare variant association. Genetic epidemiology, 38(1):21–30, 2014.

[17] Zachary R McCaw, Colm O'Dushlaine, Hari Somineni, Michael Bereket, Christoph Klein, Theofanis Karaletsos, Francesco Paolo Casale, Daphne Koller, and Thomas W Soare. An allelic-series rare-variant association test for candidate-gene discovery. The American Journal of Human Genetics, 110(8):1330–1342, 2023.

[18] Remo Monti, Pia Rautenstrauch, Mahsa Ghanbari, Alva Rani James, Matthias Kirchler, Uwe Ohler, Stefan Konigorski, and Christoph Lippert. Identifying interpretable gene-biomarker associations with functionally informed kernel-based tests in 190,000 exomes. Nature communications, 13(1):5332, 2022.

[19] Stephan Morgenthaler and William G Thilly. A strategy to discover genes that carry multi-allelic or mono-allelic risk for common diseases: a cohort allelic sums test (cast). Mutation Research/Fundamental and Molecular Mechanisms of Mutagenesis, 615(1-2):28–56, 2007.

[20] Andrew P Morris and Eleftheria Zeggini. An evaluation of statistical approaches to rare variant analysis in genetic association studies. Genetic epidemiology, 34 (2):188–193, 2010.

[21] Kiran Musunuru and Sekar Kathiresan. Genetics of common, complex coronary artery disease. Cell, 177(1):132–145, 2019.

[22] Robert M Plenge, Edward M Scolnick, and David Altshuler. Validating therapeutic targets through human genetics. Nature reviews Drug discovery, 12(8): 581–594, 2013.

[23] Alkes L. Price, Gregory V. Kryukov, Paul I.W. de Bakker, Shaun M. Purcell, Jeff Staples, Lee-Jen Wei, and Shamil R. Sunyaev. Pooled association tests for rare variants in exon-resequencing studies. The American Journal of Human Genetics, 86(6):832–838, 2010. ISSN 0002-9297. doi: https://doi.org/10.1016/j.ajhg.2010.04.005. URL https://www.sciencedirect.com/science/article/pii/S0002929710002077.

[24] Rajesh Ranganath, Sean Gerrish, and David Blei. Black box variational inference. In Artificial intelligence and statistics, pages 814–822. PMLR, 2014.

[25] Danilo Jimenez Rezende, Shakir Mohamed, and Daan Wierstra. Stochastic backpropagation and approximate inference in deep generative models. In International conference on machine learning, pages 1278–1286. PMLR, 2014.

[26] Valentine Svensson, Adam Gayoso, Nir Yosef, and Lior Pachter. Interpretable factor models of single-cell rna-seq via variational autoencoders. Bioinformatics, 36(11):3418–3421, 2020.

[27] Guhan Ram Venkataraman, Christopher DeBoever, Yosuke Tanigawa, Matthew Aguirre, Alexander G Ioannidis, Hakhamanesh Mostafavi, Chris CA Spencer, Timothy Poterba, Carlos D Bustamante, Mark J Daly, et al. Bayesian model comparison for rare-variant association studies. The American Journal of Human Genetics, 108(12):2354–2367, 2021.

[28] Michael C Wu, Seunggeun Lee, Tianxi Cai, Yun Li, Michael Boehnke, and Xihong Lin. Rare-variant association testing for sequencing data with the sequence kernel association test. The American Journal of Human Genetics, 89(1):82–93, 2011.

[29] Long-Yan Yang, Cai-Guo Yu, Xu-Hong Wang, Sha-Sha Yuan, Li-Jie Zhang, Jia-Nan Lang, Dong Zhao, and Ying-Mei Feng. Angiopoietin-like protein 4 is a high-density lipoprotein (hdl) component for hdl metabolism and function in nondiabetic participants and type-2 diabetic patients. Journal of the American Heart Association, 6(6):e005973, 2017.

[30] Yi Yang, Saonli Basu, and Lin Zhang. A bayesian hierarchically structured prior for rare-variant association testing. Genetic epidemiology, 45(4):413–424, 2021.

[31] Nengjun Yi and Degui Zhi. Bayesian analysis of rare variants in genetic association studies. Genetic epidemiology, 35(1):57–69, 2011.

[32] Wei Zhou, Wenjian Bi, Zhangchen Zhao, Kushal K Dey, Karthik A Jagadeesh, Konrad J Karczewski, Mark J Daly, Benjamin M Neale, and Seunggeun Lee. Saige-gene+ improves the efficiency and accuracy of set-based rare variant association tests. Nature genetics, 54(10):1466–1469, 2022.
